# Translation and validation of the Swedish version of the early-onset scoliosis 24-item questionnaire

**DOI:** 10.1007/s43390-025-01064-5

**Published:** 2025-03-07

**Authors:** I. Green-Petersen, T. Cheng, S. Blixt, P. Gerdhem

**Affiliations:** 1 Department of Orthopaedics and Hand Surgery, Uppsala University Hospital, Uppsala, Sweden; 2 Department of Surgical Sciences, Uppsala University, Uppsala, Sweden

**Keywords:** Early-Onset scoliosis, Early-Onset Scoliosis 24-Item Questionnaire, Scoliosis Research Society 22-Item Questionnaire revised, EuroQol-5D, Translation, Cross-Cultural validation

## Abstract

**Purpose:**

This study aimed to translate and validate the Early-Onset Scoliosis 24-Item Questionnaire (EOSQ-24) into Swedish.

**Methods:**

Following international guidelines, the EOSQ-24 was double forward translated by independent translators, reviewed by experts, and distributed to caregivers. A single back translation was performed, and the Swedish version was finalized. The Swedish EOSQ-24 was made available online for clinically active spinal surgeons to use during outpatient visits, where it was distributed to caregivers of early-onset scoliosis (EOS) patients aged 0–15 years. Internal consistency was assessed using Cronbach’s alpha, item-total correlation, and analysis of floor and ceiling effects. Convergent validity was examined using the EuroQol-5D 3 level (EQ-5D) and the Scoliosis Research Society 22-Item Questionnaire revised (SRS-22r).

**Results:**

Responses from 140 caregivers were collected. Ceiling effects ranged from 21 to 74%, with no floor effects > 15%. Internal consistency was excellent (Cronbach’s alpha = 0.9), except for two domains: “General Health” (0.6) and “Pulmonary Function” (0.4). The item–total correlation was poor for “Pulmonary Function”. EOSQ-24 domains showed significant convergent validity with SRS-22r (Spearman’s rho 0.5 to 0.8) and the EQ-5D (Spearman’s rho -0.6 to -0.8). Stepwise regression showed lower scores for patients with neuromuscular scoliosis, indicating sensitivity for scoliosis etiology.

**Conclusion:**

The Swedish EOSQ-24 is a valid, disease-specific questionnaire with excellent internal consistency suitable for use in a clinical setting. Minor inconsistencies are not unique for the Swedish translation.

**Level of evidence:** II.

## Introduction

Early-onset scoliosis (EOS) is defined as an abnormal curvature of the spine occurring before the age of 10 years [1]. The condition is heterogeneous and may be classified according to etiology and severity of the deformity [2]. If left untreated, EOS can cause cardiorespiratory failure, compromised respiratory development, and significantly increased mortality [3–5]. Treatment options include bracing, casting, and growth-friendly surgery. Goals of the treatment include obtaining and maintaining spinal deformity correction as well as achieving adequate spinal growth and lung development [6, 7]. For many years, successful treatment was based on radiological assessment. In recent years, more attention has been given to the assessment of health-related quality of life in the EOS population [8, 9]. Health-related quality of life measurement has been seen as challenging in this population due to young age, comorbidities, and population heterogeneity. In 2011, Corona et al. developed the Early-Onset Scoliosis Questionnaire, a disease-specific measure reflecting health-related quality of life in children as well as caregiver burden [10]. The questionnaire was finalized and validated by Matsumoto et al. in 2018 as the Early-Onset Scoliosis 24-Item Questionnaire (EOSQ-24), containing 24 items categorized into 11 domains [11]. The questionnaire was originally developed in English and has been translated and cross-culturally validated in a number of different languages, including Arabic, Dutch, German, Norwegian, and Persian [12–16]. However, the EOSQ-24 has not yet been translated and validated for use in the Swedish language. Our aims with this study were to translate the EOSQ-24 to the Swedish language and cross-culturally validate it in the Swedish EOS population.

## Materials and methods

### Translation, adaptation, and cross-cultural validation

Before the translation process of the EOSQ-24 into Swedish, the authors contacted the original developers of the questionnaire at Columbia University Medical Center in October 2019 and attained permission for its use. Guidelines for translation and cross-cultural adaptation, previously published by Beaton et al., were followed [17]. First, two independent authorized translators with no medical knowledge performed a forward translation of the EOSQ-24 from English into Swedish. Thereafter, a first synthesized draft was created by the senior author (PG) and distributed to three Swedish-speaking persons, two doctors and one medical secretary, for review. The draft was then adjusted accordingly by the first and senior author. The revised draft was sent out via e-mail to seven experts on spinal disease in Sweden and two Swedish-speaking spinal disease experts in Finland for comments. Generally, a positive response was received, and valuable comments were provided. Subsequently, 20 printed copies of the EOSQ-24 were distributed to caregivers of patients with EOS and they were specifically asked to anonymously give comments on the construction and semantics of the questionnaire. Several caregivers were uncertain regarding item 7, “Transfer”. Therefore, the authors had letter correspondence with Dr Hiroko Matsumoto who clarified the item’s purpose to be transfer between and access to locations in a broad sense. The translation was adjusted accordingly. Following that, a single back translation was performed of the edited Swedish version by an authorized translator with no medical knowledge. Thereafter, the back translation and the original version were given to a bilingual native American English and Swedish speaker with medical knowledge to compare the different versions and assess for any large semantic differences. Finally, after a last round of expert reviews that resulted in no further adjustments, the finalized Swedish version was prepared for validation.

### Data collection

All data in this study was collected from the Swedish Spine Registry (Swespine). Swespine has since 2007 collected patient-reported outcome data pre- and postoperatively in conjunction with scoliosis surgery using the scoliosis-specific questionnaire Scoliosis Research Society 22-Item Questionnaire revised (SRS-22r), the generic health-related quality of life questionnaire EuroQol-5D 3 level version (EQ-5D), and other questions related to health. Until now, there has been no Swedish questionnaire specifically designed for disabled children and/or those with early-onset scoliosis.

### EOSQ-24

The finalized Swedish version of the EOSQ-24 was uploaded to the Swespine official website in 2020 and made available to all clinicians in Sweden treating EOS patients in their clinical practice either preoperatively or postoperatively. The questionnaire was distributed to patients in the age group 0 through 15 years. The EOSQ-24 consists of 24 items pertaining to health-related quality of life, family burden, and satisfaction. The items are further divided into 11 domains, assessing various aspects of life affected by scoliosis, as well as evaluating family burden and satisfaction from both the child's and parent's perspectives. Each item is scored from 1 (worst) to 5 (best). The algebraic means of the answered items of each domain were transformed into standardized 0–100 scores using the following algorithm: ((algebraic mean of answered items – 1)/4)*100).

### EQ-5D

EQ-5D is a standardized, generic instrument for describing and evaluating health-related quality of life. The instrument is available in a validated Swedish version [18]. The EQ-5D consists of five domains: mobility, usual activities, self-care, pain, and anxiety. In Swespine, the British tariff is used to transform domain results into an index ranging from −0.6 (worst possible health) to 1 (best possible health). Swespine uses the adult version of EQ-5D for all ages and allows for proxy answering [19].

### SRS-22r

The SRS-22r was constructed to evaluate quality of life after surgical treatment in adolescent idiopathic scoliosis, but has been used also in non-idiopathic scoliosis [20, 21]. The questionnaire has been translated and validated in the Swedish language [22]. The SRS-22r consists of 22 questions divided into five different domains: function, pain, self-image, mental health, and satisfaction. The satisfaction domain measures postoperative treatment satisfaction. A total score is calculated as the mean of all domains. A subscore can be calculated based on the mean of four domains, in which the satisfaction domain is excluded. The SRS-22r is used for all ages in Swespine. For each question, domain, total score, and subscore, a value between 1 (worst) and 5 (best) can be obtained.

### Surgical data

Diagnosis and type of treatment was based on the Swespine data as provided by the treating surgeon.

### Respondents

A total of 140 EOS patients had questionnaires answered by their caregivers or guardians from November 2020 to July 2022. Individuals representing all types of scoliosis etiologies were included in this study. No individual answered the questionnaire more than once.

### Ethical permit

The Swespine data collection is continuously evolving and is part of routine care. The translation of the EOSQ-24 and introduction into Swespine is part of this evolution. All patients scheduled to undergo surgery are informed about the quality registry Swespine. Surgical data is collected unless the patients or caregivers actively abstain from participation, and answering the questionnaires is voluntary. Ethical permit to study outcomes in relation to scoliosis surgery in the Swedish Spine registry has previously been granted by the Regional Ethical Review Board in Stockholm, Sweden (2012/172-31/4), and by the Ethical Review Authority in Sweden (2019-00136).

### Statistical analysis of data

Scoring of the EOSQ-24 questions and domains was calculated according to the questionnaire instructions. Descriptive statistics for the individual questions included the median, first quartile, third quartile, floor and ceiling effects (the frequency of the lowest and highest score, respectively), as well as missing proportion. A threshold of > 15% frequency for the lowest or highest score was determined for floor and ceiling effects, respectively [23].

Internal consistency was evaluated using Cronbach’s alpha [24]. Cronbach’s alpha < 0.7 indicated poor, 0.7–0.8 good, and > 0.8 excellent internal consistency [13]. The “corrected item–total correlation” was used to determine the relationship between each item of the EOSQ-24 and the total score, with a value of > 0.3 indicating an acceptable relationship between the unique question and the questionnaire as a whole [23]. “Cronbach’s alpha if item deleted” was used to test the effect of removing one item on the total value of Cronbach’s alpha. Missing values were imputed with the item mean for the calculation of Cronbach’s alpha.

Convergent validity was assessed by comparing the mean domain scores of the EOSQ-24 with comparable domains in the SRS-22r and the EQ-5D questionnaires filled out at the same time point. The SRS-22r has been used in previous studies to assess construct validity for the EOSQ-24 [13, 16]. Spearman’s rank correlation coefficient (rho) was used to estimate the convergent validity. Positive or negative values of rho < 0.3, 0.3–0.6, and > 0.6 were considered low, moderate, and high correlations, respectively [25].

A multivariate analysis was performed with initial stepwise regression. Age at surgery, sex, scoliosis etiology, pre- or postoperative status, and Cobb angle of major curve were compared with mean EOSQ-24 total score.

Calculation of EOSQ-24 scores was performed in IBM SPSS (v28.0. Armonk, NY, USA). Statistical analysis was performed in R (R version 4.2.2., R Core Team (2022), R Foundation for Statistical Computing, Vienna, Austria) using RStudio (RStudio Team, PBC, Boston, MA, USA, v2022.12.0 + 353).

## Results

The clinical and demographic characteristics of the 140 included individuals are described in Table 1. Descriptive statistics of individual questions are seen in Table 2. All items in the questionnaire showed a left-skewed distribution, with median values of 4 or higher. Ceiling effects ranged from 21% (item 1, general health) to 74% (item 4, pulmonary function). No floor effects > 15% were seen. The proportion of missing answers to individual questions was low, ranging from 0 to 6%.

**Table 1:** Patient demographics

Total no. of individuals, *n*	140
Female sex, *n* (%)	94 (67%)
Mean age at operation, years (SD, range)	12 (3, 3–14)
Scoliosis etiology	
Idiopathic, *n* (%)	67 (48%)
Congenital, *n* (%)	18 (13%)
Neuromuscular, *n* (%)	23 (16%)
Other or Non-classified, *n* (%)	32 (23%)
Treatment stage at time of EOSQ-24 Completion, *n* (%)	
Preoperative*	41 (29%)
1 year postoperative	11 (8%)
2 years postoperative	53 (38%)
5 years postoperative	22 (16%)
10 years postoperative	13 (9%)
Type of surgery*	
Anterior fusion	6 (4%)
Posterior fusion	117 (84%)
Anteroposterior fusion	3 (2%)
Growth promoting	7 (5%)
Other including osteotomy and non-defined	7 (5%)
Mean preop major Cobb coronal angle, ° (SD, range) (*n* = 118)	62 (15, 15–101)
Clinically important kyphosis,° † (SD) (*n* = 19)	67 (30)

*Data for all 140 patients, among which 41 answered the questionnaires preoperatively

^†^Kyphosis was reported by the surgeon only in cases of clinical significance

**Table 2:** Descriptive statistics of the EOSQ-24

	Median score	First quartile score	Third quartile score	Floor effect, *n* (%)	Ceiling effect, *n* (%)	Missing data. *n* (%)
*General Health*						
Q1	4	3	4	4 (3%)	29 (21%)	0 (0%)
Q2	4	4	5	3 (2%)	62 (44%)	1 (1%)
Pain						
Q3	4	3	5	4 (3%)	45 (32%)	1 (1%)
Q4	4	3	5	1 (1%)	48 (34%)	1 (1%)
*Pulmonary Function*						
Q5	5	4	5	2 (1%)	101 (72%)	4 (3%)
Q6	4	3	5	6 (4%)	49 (35%)	4 (3%)
Transfer						
Q7	5	4	5	10 (7%)	95 (68%)	3 (2%)
*Physical Function*						
Q8	4	3	5	6 (4%)	67 (48%)	1 (1%)
Q9	5	4	5	13 (9%)	101 (72%)	2 (1%)
Q10	5	4	5	19 (14%)	91 (65%)	5 (4%)
*Daily Living*						
Q11	5	4	5	16 (11%)	99 (71%)	2 (1%)
Q12	5	4	5	20 (14%)	97 (69%)	3 (2%)
*Fatigue*						
Q13	4	4	5	2 (1%)	63 (45%)	2 (1%)
Q14	4	3	5	8 (6%)	48 (34%)	4 (3%)
*Emotion*						
Q15	5	4	5	3 (2%)	71 (51%)	2 (1%)
Q16	4	3	5	4 (3%)	55 (39%)	4 (3%)
*Parental Impact*						
Q17	4	3	5	8 (6%)	38 (27%)	5 (4%)
Q18	5	4	5	5 (4%)	79 (56%)	4 (3%)
Q19	4	3	5	9 (6%)	62 (44%)	4 (3%)
Q20	5	4	5	2 (1%)	87 (62%)	5 (4%)
Q21	5	4	5	2 (1%)	94 (67%)	5 (4%)
*Financial Impact*						
Q22	5	4	5	3 (2%)	80 (57%)	7 (5%)
*Satisfaction*						
Q23	4	3	5	6 (4%)	57 (41%)	3 (2%)
Q24	4	3	5	4 (3%)	61 (44%)	8 (6%)

Internal consistency was 0.9 for Cronbach’s alpha for the entire Swedish EOSQ-24 (Table 3). Two domains showed values < 0.8 for Cronbach’s alpha: General Health (0.6) and Pulmonary Function (0.4). The ‘Cronbach’s alpha If Item Deleted’ values closely approximated the overall Cronbach’s alpha for all domains, suggesting that removing any single item would not significantly improve internal consistency. However, the corrected item–total correlation revealed low correlations with the overall questionnaire for both items 5 and 6 in the’Pulmonary Function’ domain, with item 6 falling below the acceptable value (0.2).

**Table 3:** Internal consistency of the EOSQ-24

EOSQ-24 domain		Mean score	Standard deviation	Corrected item–total correlation	Cronbach’s alpha if item deleted	Cronbach’s alpha per domain
General Health		73	20			0.6
	Q1	4	1	0.6	0.9	
	Q2	4	1	0.5	0.9	
Pain		75	23			0.9
	Q3	4	1	0.5	0.9	
	Q4	4	1	0.5	0.9	
Pulmonary Function		79	22			0.4
	Q5	5	1	0.4	0.9	
	Q6	4	2	0.2	0.9	
Transfer		82	32	N/A	N/A	N/A
	Q7	4	1	0.6	0.9	
Physical Function		80	29			0.9
	Q8	4	1	0.7	0.9	
	Q9	4	1	0.7	0.9	
	Q10	4	1	0.7	0.9	
Daily Living		80	34			0.8
	Q11	4	1	0.6	0.9	
	Q12	4	2	0.6	0.9	
Fatigue		74	26			0.9
	Q13	4	1	0.7	0.9	
	Q14	4	1	0.7	0.9	
Emotion		78	24			0.8
	Q15	4	1	0.6	0.9	
	Q16	4	1	0.6	0.9	
Parental Burden		77	23			0.9
	Q17	4	1	0.7	0.9	
	Q18	4	1	0.7	0.9	
	Q19	4	1	0.8	0.9	
	Q20	4	1	0.7	0.9	
	Q21	5	1	0.7	0.9	
Financial Burden		83	25	N/A	N/A	N/A
	Q22	4	1	0.6	0.9	
Satisfaction		74	28			0.9
	Q23	4	1	0.7	0.9	
	Q24	4	1	0.7	0.9	

N/A indicates Cronbach’s alpha is not applicable for the single-item subdomain

All domains of the EOSQ-24 that were compared with the relevant domains of the SRS-22r and the EQ-5D correlated significantly when using the Spearman’s rank correlation (Table 4). Rho values for the SRS-22r ranged from 0.5 to 0.8, and for the EQ-5D from − 0.6 to − 0.8.

**Table 4:** Convergent validity of the EOSQ domains in relation with SRS-22r and EQ-5D

EOSQ-24	SRS-22r	Spearman’s rho**	EQ-5D	Spearman’s rho**
General Health	Subscore SRS-22r*	0.5	N/A	
Pain	Pain	0.8	Pain/Discomfort	− 0.6
Transfer	Function	0.5	Mobility	− 0.6
Physical Function	Function	0.7	Mobility	− 0.7
Daily Living	Function	0.5	Self-care	− 0.8
Emotion	Mental health	0.5	Anxiety/Depression	− 0.6
Financial Burden	Function	0.5	N/A	

*The subscore of the SRS-22r includes all domains of the SRS-22r except “Satisfaction”

^**^All *p* < 0.001

**Fig. 1:**
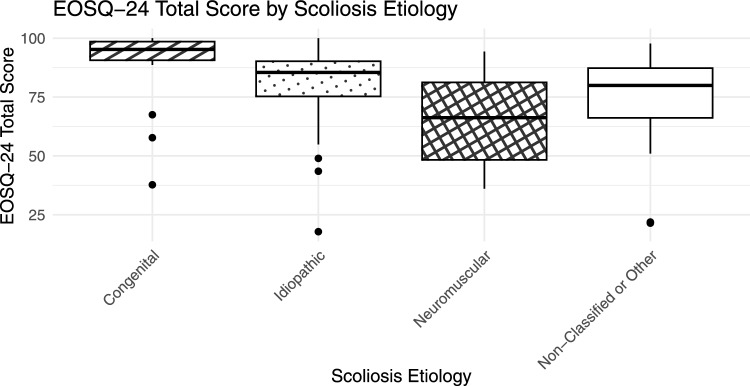
Box plots of EOSQ-24 total score by scoliosis etiology. Boxes represent the 25th to 75th percentiles, with the median as a central line. Whiskers extend to 1.5 times the interquartile range, and dots indicate outliers

Stepwise regression was performed including age at surgery, sex, scoliosis etiology, pre- or postoperative status and major curve Cobb angle at surgery as potential predictors of the total EOSQ-24 score. A final multivariate regression model showed significantly lower mean total EOSQ-24 scores for individuals with neuromuscular scoliosis (*p* < 0.001) and individuals in the group Other Etiology or Non-classified (*p* = 0.002). There was no significant correlation between the total EOSQ-24 score and age at surgery (*p* = 0.49). See Fig. 1 for a box plot of total EOSQ-24 scores by scoliosis etiology. Etiologies included in “Other or Non-classified” were metabolic (*n* = 2), neurofibromatosis (*n* = 1), Scheuermann (*n* = 1), syndromic (*n* = 1), tumor spine (*n* = 1), other (*n* = 20), and unknown (*n* = 6).

## Discussion

This study represents a cross-cultural translation and validation of the EOSQ-24 from English to Swedish, conducted in accordance with international guidelines. It marks the first cross-cultural adoption and validation of a questionnaire in Swedish specifically designed for children with early-onset scoliosis. Our results support the suitability for application of the Swedish version of the EOSQ-24 in Sweden.

### Internal consistency

The Swedish EOSQ-24 showed excellent internal consistency across most domains, comparable to previous translations [13, 14, 16]. Similar to previous cross-cultural adaptations in Dutch, Brazilian Portuguese, and traditional Chinese, lower Cronbach alpha values were observed in the domains “General Health” and “Pulmonary Function” [13, 26, 27]. The authors of the Dutch version noted a discrepancy in the “General Health” domain, with higher scores for Q2 than Q1, also seen in our study [13]. This suggests caretakers perceive the child as seldom being sick but still having poor general health. Perhaps, this is because the word “sick” often means contracting an infectious disease, whereas the child’s poor condition could be caused by their underlying diagnosis and comorbidities. In the “Pulmonary Function” domain, we noticed substantially higher scores for Q5 than Q6, meaning most respondents felt that the child often experienced shortness of breath during “activities”, but not when crying/babbling/speaking. This discrepancy between questions was also seen in the Dutch and to a lesser extent in the traditional Chinese version [13, 27]. We speculate that caretakers might interpret the word “activities” as meaning some kind of physically demanding activities, in which case it is not surprising that more children are exhausted by those activities than oral communication. Etiological heterogeneity has also been earlier proposed as a reason for the lower internal consistency in these domains by the authors of the Brazilian Portuguese EOSQ-24, which was also the case in our material [26].

### Floor and ceiling effects

Ceiling effects were seen for several questions, most notably in the domains “Pulmonary Function”, “Physical Function”, “Transfer” and “Daily Living”. In “Pulmonary Function”, item 5 carried the ceiling effect, and the domain also had a low Cronbach’s alpha as discussed earlier [13, 27]. Comments during the translation process indicated Q7, “Transfer”, was unclear on whether it referred to body movement or access to places. Dr Matsumoto clarified it was meant to be inclusive of both. The ceiling effect for Q7 may result from the accessibility of buildings in Sweden, accommodating physical disabilities, including larger electronic wheelchairs. Ceiling effects for “Transfer” and the other items might also be related to early symptomatic treatment and a standardized national follow-up program in Sweden for cerebral palsy. A non-response bias might also be present, with respondents’ children possibly in better health than non-respondents’.

### Convergent validity

The Swedish EOSQ-24 showed moderate to high correlations with the SRS-22r and the EQ-5D in relevant domains. The highest correlation was in the “Pain” domain with the corresponding domain of the SRS-22r, similar to the Persian EOSQ-24 [16]. The Dutch version also showed similar validity with the SRS-22r, but had higher correlations for “Transfer”, “Physical Function”, and “Daily Living”. It is noted that in the Dutch study, parents completed both questionnaires [13]. A 2024 comparative study of the EOSQ-24 and the SRS-22r by Gottlieb et al. showed similar results to the Swedish EOSQ-24, *r* = 0.8 for “Pain”, but lower correlations in other domains [28]. For the EQ-5D, the strongest correlation was between “Daily Living” and “Self-care”, indicating that the Swedish EOSQ-24 assessment lies close to the children’s own perception of daily functioning.

### Discriminative ability

The Swedish EOSQ-24 showed significantly lower scores for individuals with neuromuscular scoliosis and in the category “Other or Non-classified”, indicating sensitivity to scoliosis etiology similar to previous translations [13, 14]. There were no significant differences between pre- and postoperative patients, suggesting the Swedish EOSQ-24 is suitable for both preoperative assessment and postoperative follow-up. Unlike previous studies, we did not observe any discriminative ability based on Cobb angles [12, 14, 16, 29]. We believe this is likely due to the small number of patients with Cobb angles < 30° in our cohort (only 2 out of 118 with available data).

### Strengths and limitations

This study included 140 individuals, the largest cohort among EOSQ-24 translations [12–16, 26, 27, 29, 30]. Translation was conducted by professional translators and reviewed by national experts. The cohort included both preoperative and postoperative patients with significant scoliosis (mean Cobb angle > 60°). A limitation is the absence of test–retest analysis, preventing the calculation of the intraclass correlation coefficient. This is due to data being collected by multiple centers at planned outpatient visits, where repeated administration within a short time frame was not feasible. We allowed patients up to the age of 15 years to answer the questionnaire. Since this is a surgical population, we estimated that the majority of the patients had their scoliosis diagnosis before the age of 10 years.

### Linguistic considerations

Swedish is spoken primarily in Sweden, with a relatively homogeneous dialect. We do not believe that there should be any local differences in interpreting the Swedish EOSQ-24 in Sweden. The Swedish language is also spoken in parts of Finland. However, all our respondents are residents in Sweden and we cannot claim to have validated the Swedish EOSQ-24 for a Finnish population.

## Conclusion

The Swedish EOSQ-24 is a valid, disease-specific patient-reported outcome measure for EOS patients. It is suitable for use in a clinical setting and for further studies on quality of life in EOS patients. A few imperfections were noted in the internal consistency, but these were not unique to the Swedish version.

## Data Availability

The data that was used to conduct this study are not openly available due to reasons of sensitivity. Data are located in controlled access data storage at Uppsala University. Reasonable requests for accessing the data should be directed to the corresponding author.
